# Peripheral Nerve Ultrasound for the Differentiation between ALS, Inflammatory, and Hereditary Polyneuropathies

**DOI:** 10.3390/medicina59071192

**Published:** 2023-06-24

**Authors:** Annkatrin Hildebrand, Frank Schreiber, Luisa Weber, Philipp Arndt, Cornelia Garz, Susanne Petri, Johannes Prudlo, Sven G. Meuth, Yannic Waerzeggers, Solveig Henneicke, Stefan Vielhaber, Stefanie Schreiber

**Affiliations:** 1 Department of Neurology, Otto von Guericke University Magdeburg (OVGU), 39120 Magdeburg, Germany; 2 German Center for Neurodegenerative Diseases (DZNE), 39120 Magdeburg, Germany; 3 Department of Neurology, Saarland University Medical Center, 66421 Homburg, Germany; 4 Leibniz Institute for Neurobiology (LIN), 39120 Magdeburg, Germany; 5 Department of Neurology, Hannover Medical School, 30625 Hannover, Germany; 6 Department of Neurology, Rostock University Medical Center, 18147 Rostock, Germany; 7 German Center for Neurodegenerative Diseases (DZNE), 18147 Rostock, Germany; 8 Department of Neurology, Medical Faculty, Heinrich Heine University, 40225 Düsseldorf, Germany; 9 Center for Behavioral Brain Sciences (CBBS), 39120 Magdeburg, Germany; 10 Center for Intervention and Research on Adaptive and Maladaptive Brain Circuits Underlying Mental Health (C-I-R-C), Jena-Magdeburg-Halle, Germany

**Keywords:** peripheral nerve ultrasound, tibial nerve, amyotrophic lateral sclerosis, inflammatory neuropathy, hereditary neuropathy, cross-sectional area, nerve microvascular blood flow

## Abstract

*Background and Objectives:* Ultrasound (US) is a non-invasive tool for the in vivo detection of peripheral nerve alterations. *Materials and Methods:* In this study, we applied nerve US to assist the discrimination between the spectrum of amyotrophic lateral sclerosis (ALS, *n* = 11), chronic inflammatory demyelinating polyradiculoneuropathy (CIDP, *n* = 5), and genetically confirmed Charcot–Marie–Tooth disease (CMT, *n* = 5). All participants and *n* = 15 controls without neurological diseases underwent high-resolution US of the bilateral tibial nerve. The nerve cross-sectional area (CSA) and nerve microvascular blood flow were compared between the groups and related to cerebrospinal fluid (CSF) measures, clinical symptoms, and nerve conduction studies. The analyses are part of a larger multimodal study on the comparison between US and 7 Tesla (7T) magnetic resonance neurography (MRN). *Results:* The patients and controls were matched with respect to their demographical data. CMT had the longest disease duration, followed by CIDP and ALS. CSA was related to age, weight, and disease duration. CSA was larger in CMT and CIDP compared to ALS and controls. The blood flow was greatest in CIDP, and higher than in CMT, ALS, and controls. In ALS, greater CSA was correlated with greater CSF total protein and higher albumin quotient. The US measures did not correlate with clinical scores or nerve conduction studies in any of the subgroups. *Conclusion:* Our results point towards the feasibility of CSA and blood flow to discriminate between ALS, CIDP, and CMT, even in groups of small sample size. In ALS, larger CSA could indicate an inflammatory disease subtype characterized by reduced blood–nerve barrier integrity. Our upcoming analysis will focus on the additive value of 7T MRN in combination with US to disentangle the spectrum between more inflammatory or more degenerative disease variants among the disease groups.

## 1. Introduction

In ultrasound (US), the nerve cross-sectional area (CSA) has been used for several years and has evolved as a diagnostic marker, particularly in compression or peripheral neuropathies. Both chronic inflammatory demyelinating polyradiculopathy (CIDP) and Charcot–Marie–Tooth disease (CMT) are commonly related to CSA enlargement, but depending on disease stage and genetic background, unaltered CSA values have been found as well [1]. For example, untreated CIDP patients exhibit larger CSA, probably indicative of greater disease activity, than treated patients [2].

In amyotrophic lateral sclerosis (ALS), CSA heterogeneity is even greater, and patients show reduced, unaltered, or enlarged CSA, hampering the translation of nerve US as a pure diagnostic marker into the clinic [3,4,5,6,7,8].

Nerve microvascular blood flow seems to have the potential to indicate inflammation and could be a valuable pathophysiological marker across disease entities [9,10,11].

This study investigates the CSA and nerve microvascular blood flow of the tibial nerve in a small cohort of ALS, CIDP, and CMT patients who are part of a larger multimodal study applying fusion imaging between nerve US and ultra-high-resolution 7 Tesla (7T) magnetic resonance neurography (MRN). Ultrasound markers were set in relation to nerve conduction study, cerebrospinal fluid (CSF) parameters, and clinical diagnostics, with a particular focus on the identification of subtypes within the disease entities.

## 2. Materials and Methods

### 2.1. Ethical Approval

The study was approved by the ethics committee of the Otto von Guericke University Magdeburg (No 16/17 13.02.2017 plus addendum 16 March 2018) and all study participants gave written informed consent.

### 2.2. Patients and Subjects

#### 2.2.1. Recruitment

The recruitment of ALS, CIDP, and CMT patients took place from June 2018 to January 2022 as part of a larger study of fusion imaging from 7T MRN and high-resolution ultrasound in the neuromuscular outpatient clinics of the Departments of Neurology from the Otto von Guericke University Magdeburg, the Hannover Medical School, and the Rostock University Medical Center, Germany.

Age- and sex-matched healthy controls without any neurological or neuromuscular disorder and without neurological symptoms (e.g., tingling paresthesia, paresis, muscle atrophy) were taken from an already existent pool of the Department of Neurology, Magdeburg [8,12].

#### 2.2.2. Inclusion and Exclusion Criteria

ALS patients were included based on the revised El-Escorial criteria with definite, probable, and possible disease [13]. CIDP patients were included based on the EFNS criteria with appropriate clinical symptoms, nerve conduction studies, and typical cerebrospinal fluid (CSF) findings [14]. Prerequisites for the study participation in the group of hereditary neuropathies were typical symptoms and nerve conduction studies, as well as a positive molecular genetic finding for a hereditary neuropathy [15].

The exclusion criteria were unconfirmed or incomplete diagnoses and secondary diagnoses that made a clear assignment of neurological symptoms to one of the included diagnoses (ALS, CIDP, or CMT) difficult or impossible: monoclonal gammopathy of undetermined significance (MGUS) with neuropathic symptoms, poorly controlled diabetes mellitus, positive alcohol and medication history with a suspicion of toxic neuropathy, or neurological diagnoses with severe impairment of the lower extremity (stroke, cerebral hemorrhage, cerebral tumor, peripheral nerve damage due to trauma).

Because the study is part of a larger study with 7T MRN, the exclusion criteria for measurements in 7T magnetic resonance imaging had to be additionally considered: impaired breathing in the supine position; respiratory support (noninvasive ventilation or tracheostoma); percutaneous endoscopic gastrostomy tube; ferromagnetic implants, e.g., screws/plates for osteosynthesis, joint endoprostheses, or dental implants (without available evidence of 7T MR suitability); implanted electrical devices, e.g., pacemakers, cochlear implants, drug pumps, cerebral or spinal cord stimulation electrodes; large tattoos, especially in the area of the lower leg; presence of tinnitus; pregnancy and lactation; pronounced claustrophobia.

**Figure 1:**
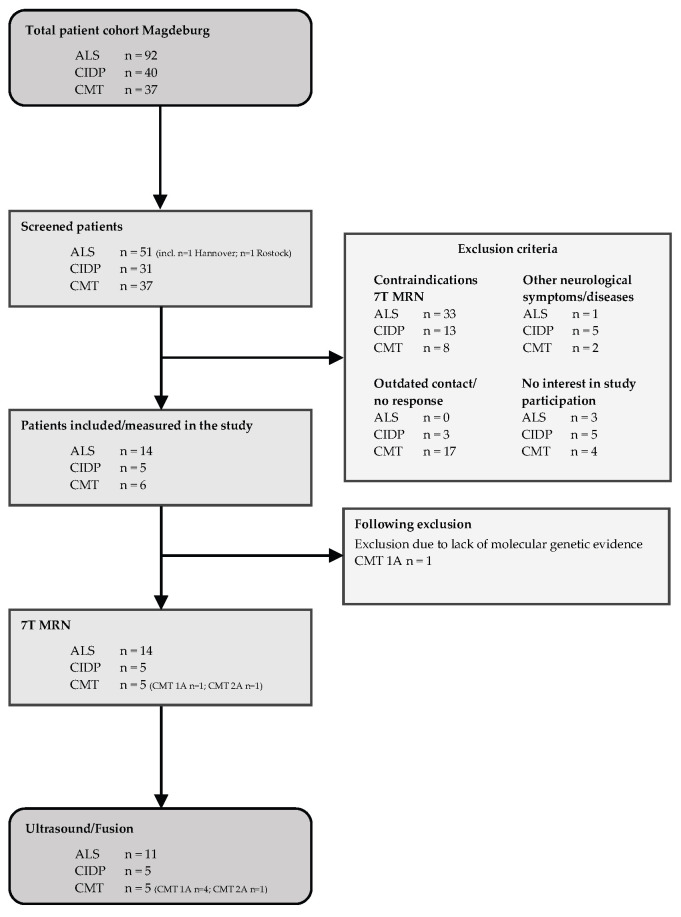
Patient recruitment flowchart.

Shown in Figure 1 are the individual recruitment processes, each divided into the diagnoses (ALS, CIDP, and CMT), starting with the total cohort in Magdeburg, followed by the screened patients in the period from June 2018 to January 2022; the measured patients; the number of patients whose data will be included in the 7T MRN analyses; and, finally, the number of patients with ultrasound data we considered for this study. The right-hand side lists the respective exclusion criteria in the trial.

#### 2.2.3. Number of Participants

According to the above criteria, 11 ALS patients, 5 CIDP patients, and 5 patients with hereditary neuropathy (4 patients with CMT 1A and 1 patient with CMT 2A), as well as 15 healthy subjects were able to be included in the study (Figure 1). Three participants (one CMT patient and two controls) had unilateral ultrasound measures only.

### 2.3. CSF Data

To determine blood–nerve barrier leakage, the total CSF protein concentration (TPC) and albumin quotient (QAlb) were included from 10 ALS and 4 CIDP patients. The timespan between lumbar puncture and US was −4.10 (−0.07 to −109.93) months.

### 2.4. Quantification of Clinical Symptoms

The clinical symptoms in ALS patients were assessed using the ALS Functional Rating Scale/revised (ASLFRS/R) and the statistical analysis took into account the total score (in 12 different categories: speech, salivation, swallowing, handwriting, feeding, dressing/personal hygiene, turning in bed, walking, climbing stairs, dyspnea, orthopnea, and respiratory insufficiency. Each ranged from 0 points = severely impaired/impossible to 4 points = normal/unimpaired) and included a gross motor function sub-score (turning in bed, walking, climbing stairs, 0–12 points) [16].

The clinical symptoms in patients with CIDP were quantified using the overall neuropathy limitation scale (ONLS) and statistics were run for the total and the sub-score grading of leg disability (Arms grade score: 0 = normal to 5 = disability of both arms prevents all purposeful movements, Legs grade score: 0 = walking/climbing stairs/running unimpaired to 7 = confined to wheelchair or bed, no purposeful movements of legs possible) [17].

In patients with CMT disease, the clinical symptoms were assessed using the CMT Neuropathy Score (CMTNS), which is divided into nine different categories (subjective sensory and motor symptoms, pin sensibility, vibration, arm and leg strength, electrophysiological measurement of the ulnar nerve), each assigned 0–4 points. Overall, a distinction is made between the following ranges: 0–10 points = mildly affected, 11–20 = moderately affected, 21–36 = severely affected. In addition, a modified sub-score to quantify lower extremity limitations (subjective sensory and motor symptoms of the legs, pin sensibility, vibration, leg strength; 0–20 points) was employed [18].

Using the Medical Research Council (MRC)-Sum score (0–60 points), the muscle strength grades of the upper/lower extremities and, separately, of the lower extremities (0–30 points) were determined in patients with polyneuropathy (CIDP and CMT) [19]. The clinical assessment of the symptoms, scoring, and US measurement were performed on the same day.

### 2.5. Nerve Conduction Studies

Bilateral electrophysiology was available for all CIDP and CMT patients. For the statistical analysis, motor tibial nerve conduction velocity and amplitudes were considered. The time interval between the nerve conduction study and the US measurement was −1.10 (0.00 to −7.50) months.

### 2.6. Imaging

#### 2.6.1. High-Resolution Nerve Ultrasound

The nerve US was performed using a Philips Medical System, Affiniti 70G, with an eL18-4 18-MHz broadband ultrasound probe. For this purpose, the study participants lay on an examination couch. B-mode images of bilateral tibial nerves were documented six times per side and the tibial nerve microvascular blood flow was recorded in three videos per side. The measurements took place at a commonly investigated anatomical location [20], proximal to the tarsal tunnel at the medial malleolus before branching into the plantar nerves, and were conducted by an experienced investigator (S.S.) blinded against electrophysiology, CSF, and clinical patient data. The choice of the anatomical localization and, in particular, the distal tibial nerve resulted from the fact that it is easily detected by the knee coil used in 7T MRN and that fusion between MRN/ultrasound is readily possible and subsequently easy to implement in clinical practice [21].

#### 2.6.2. Analysis of the Image Material

Digital Imaging and Communications in Medicine (DICOM) US images were analyzed offline by a second investigator (A.H.), not blinded against the patient data.

The tibial nerve CSA was manually delineated without the hyperechogenic epineurium using MANGO (multi-image analysis GUI) [22].

The microvascular blood flow was visually quantified as follows: grade 0: no blood flow; grade 1: 1 or 2 focal color-encoded spots; grade 2: 1 linear color-encoded line or >2 focal color-encoded spots; grade 3: >1 linear color-encoded line [23]. Therefore, the video footage (30 s) of microvascular blood flow measurement by power Doppler was converted into 268 frames, and every 10th frame was determined visually so that the neuronal blood flow was averaged from a total of 26 individual measures per side.

### 2.7. Statistics

IBM SPSS for Windows, version 28 (IBM Corp., Armonk, NY, USA) was used for the statistical analysis. Normal distribution was assessed through a Shapiro–Wilk test. For subgroup comparisons, a *t*-test, Mann–Whitney U test, and chi-square test were used for demographics, while one-way ANOVA and Kruskal–Wallis tests, both with post hoc Bonferroni adjustment, were applied for the US data. Pearson’s and Spearman’s rank bivariate correlations were adopted to relate the US with the electrophysiology, CSF, and clinical data in each diagnostic group, respectively. The median split was used to divide the disease groups into severe and mild clinical subgroups, respectively, and then compared with respect to imaging parameters using a *t*-test or Mann–Whitney U test to distinguish possible inflammatory subgroups.

The intra- and interrater reliabilities for the US measures were determined through the intraclass correlation coefficient (ICC). For this purpose, five measurement series for CSA and microvascular blood flow (each left and right sides) were randomly selected from the entire cohort. ICC was very good to excellent for CSA (intra-rating 0.981, inter-rating 0.976) and blood flow (0.908; 0.816). The interrater (L.W.) was completely blinded against diagnosis and any clinical data.

## 3. Results

The demographical data are summarized in Table 1. The subgroups did not differ with regard to age, height, weight, and sex. CMT patients had the longest disease duration, followed by CIDP and ALS patients. The US and electrophysiology measures did not differ between the left and right limbs (Supplementary Tables S1 and S2) and were averaged for further analysis. When considering the entire cohort, greater CSA was related to younger age, greater weight, and longer disease duration (Table 2). Microvascular blood flow did not correlate with any of the demographics, nor with disease duration (Table 2). A positive correlation was shown for CSA and blood flow, with larger CSA values relating to higher microvascular blood flow (rho = 0.375; *p* = 0.024). Therefore, in all further analyses, microvascular blood flow was normalized for CSA in order not to assume falsely high values for microvascular blood flow due to large CSA values.

**Figure 2:**
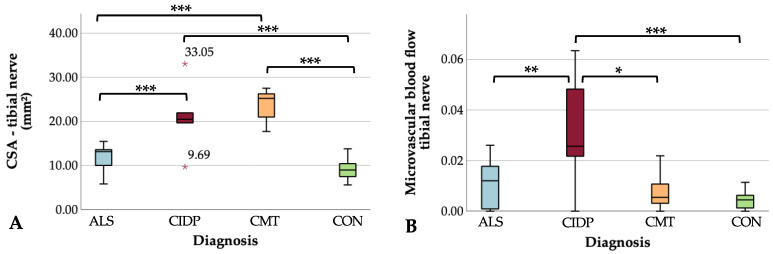
Box plots of the cross-sectional area and microvascular blood flow—tibial nerve. (**A**) The CMT group (orange) with the largest CSA followed by the CIDP cohort (red) show significantly larger CSA values of the tibial nerve than the ALS (blue) and control cohorts (green), with the latter having the smallest CSA. (**B**) Patients with CIDP (red) have significantly higher microvascular blood flow in the tibial nerve than the other three subgroups: ALS (blue), CMT (orange), and controls (green). Median (horizontal bars), 25th to 75th quartile (box), range (whiskers) and outlier >3 times the interquartile range (red asterisks) are shown. * *p* ≤ 0.05; ** *p* ≤ 0.01; *** *p* ≤ 0.001. ALS, amyotrophic lateral sclerosis; CIDP, chronic inflammatory demyelinating polyradiculopathy; CMT, Charcot–Marie–Tooth disease; CON, controls; CSA, cross-sectional area.

**Figure 3:**
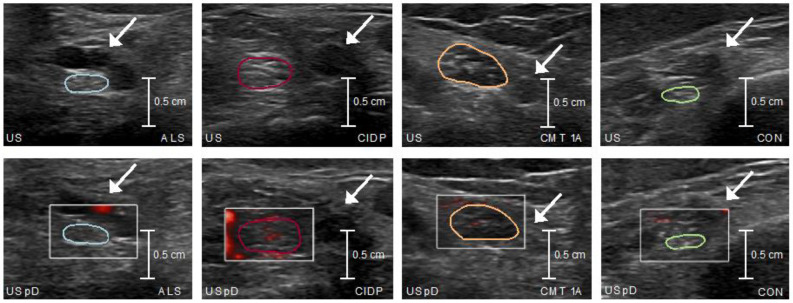
Ultrasound and ultrasound power Doppler—tibial nerve. (**Top row**) Representative B-mode ultrasound image of each subgroup. CSA of the tibial nerve is massively larger in the CMT (orange) and the CIDP (red) than in the ALS (blue) or the control (green) patients. (**Bottom row**) Microvascular blood flow at the same locations as in the top row images. CIDP (red) shows strengthened microvascular blood flow with the most red spots compared to ALS (blue), CMT (orange), and control (green). Color-coded tibial nerve cross-sectional areas; arrows indicate posterior tibial artery. US, ultrasound; US pD, ultrasound power Doppler; ALS, amyotrophic lateral sclerosis, CIDP, chronic inflammatory demyelinating polyradiculopathy, CMT, Charcot–Marie–Tooth disease; CON, controls.

Comparing the US measurements among the subgroups, CSA was significantly larger in CMT and CIDP compared to ALS and controls, and microvascular blood flow was greatest in the CIDP cohort and higher than in CMT, ALS, and controls (Table 3 and Figure 2 and Figure 3. The CSA results remained unchanged after outlier exclusion. Similarly, subdividing the ALS cohort into subtypes and comparing the imaging parameter with each other (ALS-bulbar vs. ALS-limbs; ALS-lower limbs vs. ALS-upper limbs) did not yield a significant result (Supplementary Table S3). After the exclusion of the CMT 2A case from the CMT group, comparable results were obtained for the ultrasound parameters (Supplementary Tables S4–S6). In ALS, CSA showed a large effect size positive correlation with CSF QAlb and with CSF TPC on a trend level (Table 4). Comparing ALS patients with blood–nerve barrier leakage (according to QAlb > 8 × 10^−3^ [24]) against those without leakage, CSA was slightly larger in the first group (trend level). There were no associations between microvascular blood flow and CSF parameters (Table 4 and Supplementary Figure S1). In CIDP, no correlation between CSF data (TPC, QAlb) and US measures emerged (Table 4).

When comparing clinical symptoms using adapted scores and imaging parameters (CSA, microvascular blood flow), there was a trend-level positive correlation between microvascular blood flow and the ALSFRS/R total and ALSFRS/R gross-motor sub-score in ALS, i.e., a greater flow was related to better-preserved motor function (Table 5).

In CIDP and CMT, the US data did not correlate with any of the clinical or electrophysiological data (Table 5 and Table 6). After subdividing the respective disease groups (ALS, CIDP, and CMT) into mildly or severely affected based on the clinical scores, we could not demonstrate any differences in the imaging parameters (Supplementary Tables S7–S9).

## 4. Discussion

Here, we show that the tibial nerve CSA and microvascular blood flow differ between CIDP, CMT, and ALS. The CIDP patients showed enlarged CSA and the greatest microvascular blood flow, while the CMT patients had enlarged CSA without increased flow, and in ALS, neither US parameter differed from the controls. Interestingly, we found an ALS subgroup with larger CSA values that has blood–nerve barrier leakage and probably better-preserved motor function.

Our results replicate previous findings reporting group differences for CSA and flow [10,25,26,27]. The relationship between CSA and body weight is quite well accepted [27,28,29]. An inverse correlation between CSA and age has, on the contrary, rarely been reported [27,28,30]. In our study, this relationship could be best explained by the large CSA in CMT, who had the largest CSA and the youngest age. We could further show that these US markers differ between peripheral neuropathies, ALS, and controls in even very small-sized samples and—for flow—by just applying a simple semi-quantitative approach, supporting the parameters’ diagnostic robustness. While CSA enlargement and microvascular blood flow in CIDP and CMT have mainly been studied in the proximal and upper limb nerves, there has been only rarely a focus on the distal tibial nerve [1,10,25,26,27,31,32]. With the exception of one study, likewise uncovering unaltered nerve blood flow, to the best of our knowledge, little is known about nerve microvascular flow in ALS so far [33].

In this independent cohort, we replicated our previous results and those of others reporting an ALS subgroup with larger CSA and, probably, increased nerve microvascular flow in the face of blood–nerve barrier leakage, supposedly pointing towards the existence of an inflammatory disease subtype [6,8,34]. Of clinical relevance is the hint that this subtype might display sustained motor function, and—most likely—better long-term outcomes [8]. Ultrasound could be a very valuable (and relatively unique) bedside tool to identify these patients in the clinic. Thus, the potential of nerve US goes far beyond distinguishing ALS from ALS-mimicking neuropathies and may have additional prognostic value. Similarly, in CIDP, CSA and blood flow are becoming established as markers for therapeutic decision making and monitoring [2,9,35,36]. In CMT, US (and particularly blood flow) could also aid in the in vivo stratification of accompanying nerve inflammation and disease overlaps between CMT and CIDP and could support a refined disease management in peripheral hereditary neuropathies [37]. This exciting field of the spectrum of peripheral inflammation and degeneration demands in-depth elaboration and rethinking of disease classification in a multicenter design.

The lack of correlation between CSA and electrophysiological scores in CMT and CIDP, as well as the lack of correlation between CSA and clinical symptoms in all three disease subgroups (ALS, CIDP, and CMT), are most likely explained by the small case numbers. Especially for ALS and CIDP, similar results have been shown in the literature [6,8,33]. The missing correlation between ultrasound and electrophysiology might also be explained by the different focus of the two examination techniques. Whereas ultrasound is used to study the focal morphology of a nerve, electrophysiology measures nerve functionality [6]. Moreover, peripheral nerve degeneration, i.e., smaller CSA, or peripheral nerve inflammation, i.e., greater CSA, might not necessarily explain the entire variance in nerve function and clinical status. Other limitations of our study include investigators not being fully blinded, not all lifestyle factors being covered in a controlled manner, and different time intervals between the ultrasound measurement and electrophysiology or lumbar puncture for the patients, making a clear comparison difficult. Due to the small sample size, it was difficult to subdivide the patient cohorts according to symptom onset (bulbar, upper or lower limb) in ALS and genetic subtypes in CMT and to make usable calculations, which would be of great relevance to make more specific analyses and stronger statements. In this regard, and in addition to a larger sample size, comparable sizes of patient groups and comparisons with diagnoses that mimic one another—clinically and in regard to CSA values—such as spinal muscular atrophy (SMA), especially type 4, as well as multifocal motor neuropathy (MMN) or even hereditary neuropathies with only moderately increased CSA levels (e.g., CMT 2) would also be of notable value. Structured imaging studies of multiple distal nerves, not limited to the tibial nerve, as well as in relation to proximally located nerve segments, could provide further important results. Nevertheless, with very small sample sizes, we could show the differences between ultrasound parameters that support their robustness and should be followed up in further analyses.

However, the lack of correlation might be superable by the establishment and application of additional US markers, better reflecting the already existent axonal damage and nerve degeneration (e.g., grayscale markers or fascicle measures). Future studies should focus on (i) the validation of CSA and nerve microvascular blood flow as parameters of inflammation by combining these measures with inflammatory peripheral blood biomarkers and (ii) the predictive/prognostic value of these US parameters for the clinic.

Our upcoming analysis will take into account the combination of multimodal measures such as US CSA and microvascular blood flow with 7T MRN data, e.g., fascicular T2 intensity and area, to classify the disease subgroups along the spectrum of nerve inflammation and degeneration down to and based on the pathology level.

## 5. Conclusions

We have demonstrate that US CSA and microvascular blood flow are robust diagnostic markers distinguishing neuropathies and ALS even in very small-sized samples. CSA and flow may have the potential to uncover an ALS disease subtype with more pronounced peripheral nerve inflammation and better sustained clinical function. Our upcoming studies will take advantage of multimodal fusion imaging between US and MRN to disentangle the spectrum of neuroinflammation to neurodegeneration across the disease groups of peripheral neuropathies and ALS, which will prospectively allow a pathophysiological-based classification to refine therapeutic decision making.

## Figures and Tables

**Table 1:** Demographic and clinical data.

Biometric Data	ALS (*n* = 11)	CIDP (*n* = 5)	CMT (*n* = 5)	Total Patient Cohort (*n* = 21)	Controls (*n* = 15)	*p*-Value
Age (years)	61.7 ± 9.5 61 (48–77)	56.2 ± 7.3 55 (48–67)	29.8 ± 8.6 29 (22–44)	52.8 ± 15.8 54 (22–77)	57.6 ± 22.6 69 (23–78)	U = 113.5; Z = −1.141; *p* = 0.160
Gender (male (%))	7 (64%)	3 (60%)	4 (80%)	14 (67%)	7 (47%)	Χ^2^(1) = 1.440; *p* = 0.230
Height (cm)	171.6 ± 10.3 175 (158–187)	174.2 ± 6.4 175 (165–180)	180.0 ± 16.9 176 (165–204)	174.2 ± 11.4 175 (158–204)	174.4 ± 12.0 176 (156–194)	T(32) = −0.047; *p* = 0.963
Weight (kg)	77.6 ± 17.5 77 (47–108)	86.6 ± 19.7 90 (57–111)	81.6 ± 13.1 90 (65–93)	80.7 ± 16.7 86 (47–111)	77.1 ± 16.3 73 (56–120)	T(33) = 0.639; *p* = 0.527
Disease duration (months)	18.1 ± 49.3 0.2 (0.0–165.9)	58.9 ± 47.6 35.1 (20.6–134.3)	190.6 ± 59.4 188.6 (121.3–268.5)	---	Na	H(2) = 13.181; *p* = 0.001 *

Data presented as mean ± standard deviation and median (range), except for gender. ALS, amyotrophic lateral sclerosis; CIDP, chronic inflammatory demyelinating polyradiculopathy; CMT, Charcot–Marie–Tooth disease; Na, not applicable. Total patient cohort = ALS + CIDP + CMT; *p*-values indicate significance levels for comparisons between the total patient group and controls. * *p* ≤ 0.05 (statistically significant).

**Table 2:** Correlation between ultrasound, demographic, and clinical data.

Total Cohort (*n* = 36)	Age	Gender	Height	Weight	Disease Duration
CSA	−0.539 (<0.001) **	X^2^(35) = 36.000 *p* = 0.422	0.285 (0.102)	0.450 (0.007) **	0.612 (0.003) **
Microvascular blood flow	0.038 (0.827)	X^2^(22) = 22.057 *p* = 0.456	−0.095 (0.591)	−0.287 (0.095)	−0.024 (0.917)

Data presented as Spearman’s rank correlation coefficient (rho) (*p*-value), except for gender (chi-square *p*-value); CSA, cross-sectional area; ** *p*-value considered significant after Bonferroni adjustment (*p* ≤ 0.05/2 = 0.025).

**Table 3:** Group comparison ultrasound data.

US Data	ALS (*n* = 11)	CIDP (*n* = 5)		CMT (*n* = 5)		CON (*n* = 15)	
CSA (mm^2^)	11.79 ± 2.97 (5.82–15.47)	20.97 ± 8.31 (9.69–33.05)		23.55 ± 4.07 (17.73–27.52)		9.15 ± 2.15 (5.61–13.80)	
Microvascular blood flow	0.0104 ± 0.0099 (0.00–0.03)	0.0318 ± 0.0246 (0.00–0.06)		0.0082 ± 0.0086 (0.00–0.02)		0.0044 ± 0.0037 (0.00–0.01)	
US data	*p*-value (ANOVA)	ALS vs. CIDP	ALS vs. CMT	ALS vs. CON	CIDP vs. CMT	CIDP vs. CON	CMT vs. CON
CSA (mm^2^)	F(3) = 23.63; *p* < 0.001	<0.001	<0.001	0.607	1.000	<0.001	<0.001
Microvascular blood flow	F(3) = 7.837; *p* < 0.001	0.006	1.000	1.000	0.012	<0.001	1.000

Data presented as mean ± standard deviation, (range); US, ultrasound; ALS, amyotrophic lateral sclerosis; CIDP, chronic inflammatory demyelinating polyradiculopathy; CMT, Charcot–Marie–Tooth disease, CON, controls; CSA, cross-sectional area. One-way ANOVA and post hoc Bonferroni were used. Significance was set at *p* ≤ 0.05. Significant values are shown in bold.

**Table 4:** Correlation ultrasound data vs. cerebral spinal fluid parameters.

	ALS (*n* = 10)					CIDP (*n* = 4)	
	ALS Total (*n* = 10)		Blood–Nerve Barrier Leakage				
	CSF-TPC	Qalb	YES (*n* = 5)	NO (*n* = 5)	*p*-Value	CSF-TPC	Qalb
CSA	0.655 (0.040)	0.787 (0.012) **	13.17 ± 2.072 13.21 (10.90–15.47)	11.01 ± 3.24 9.16 (5.82–13.39)	T(8) = −1.839; *p* = 0.103	0.029 (0.971)	0.021 (0.979)
Microvascular blood flow	0.152 (0.675)	0.071 (0.856)	0.0129 ± 0.0118 0.0142 (0.00–0.03)	0.0100 ± 0.0086 0.0120 (0.00–0.02)	T(8) = −0.445; *p* = 0.668	0.406 (0.594)	0.407 (0.593)

Data presented as Pearson correlation coefficient (r) (*p*-value), ** *p*-value considered significant after Bonferroni adjustment (*p* ≤ 0.05/4 = 0.0125), except for ALS (blood–nerve barrier leakage)—data presented as mean ± standard deviation and median (range), *p* ≤ 0.05/2 = 0.025 (statistically significant). ALS, amyotrophic lateral sclerosis; CIDP, chronic inflammatory demyelinating polyradiculopathy; CSA, cross-sectional area; CSF, cerebral spinal fluid; TPC, total protein concentration; QAlb, quotient albumin.

**Table 5:** Correlation ultrasound data vs. clinical scores.

Correlation	ALS (*n* = 11)		CIDP (*n* = 5)				CMT (*n* = 5)			
	ALSFRS/R Total	ALSFRS/R Gross-Motor	ONLS Total	ONLS Leg Score	MRC-Sum-Score	MRC- Lower Limbs	CMTNS Total	CMTNS Leg Score	MRC-Sum-Score	MRC- Lower Limbs
CSA	0.174 (0.609)	0.185 (0.586)	0.505 (0.385)	0.427 (0.474)	−0.538 (0.349)	−0.585 (0.301)	−0.284 (0.643)	−0.450 (0.447)	0.195 (0.753)	0.057 (0.928)
Microvascular blood flow	0.544 (0.083)	0.530 (0.093)	−0.026 (0.967)	0.032 (0.959)	0.081 (0.896)	0.140 (0.823)	0.200 (0.747)	−0.344 (0.571)	−0.108 (0.862)	−0.044 (0.944)

Data presented as Pearson correlation coefficient (r) (*p*-value), ** *p*-value considered significant after Bonferroni adjustment (i.e., ALS *p* ≤ 0.05/4 = 0.0125; CIDP and CMT *p* ≤ 0.05/8 = 0.00625). ALS, amyotrophic lateral sclerosis; CIDP, chronic inflammatory demyelinating polyradiculopathy; CMT, Charcot–Marie–Tooth disease; CSA, cross-sectional area; ALSFRS/R, ALS Functional Rating Scale/revised; ONLS, overall neuropathy limitation scale; MRC-sum-score, Medical Research Council sum score; CMTNS, CMT neuropathy score.

**Table 6:** Correlation ultrasound data vs. nerve conduction study—tibial nerve.

Correlation	CIDP (*n* = 5)			CMT (*n* = 5)		
	CMAP Ankle	CMAP Popliteal Fossa	NCV	CMAP Ankle	CMAP Popliteal Fossa	NCV
CSA	−0.798 * (0.106)	−0.949 (0.014)	−0.707 * (0.182)	0.455 * (0.441)	0.700 (0.188)	0.331 * (0.586)
Microvascular blood flow	0.573 * (0.312)	0.474 (0.420)	0.288 * (0.638)	−0.061 * (0.922)	0.500 (0.391)	−0.662 * (0.223)

Data presented as Spearman’s rank correlation coefficient (rho) (*p*-value), * Pearson correlation coefficient (r) (*p*-value), *p*-value considered significant after Bonferroni adjustment (*p* ≤ 0.05/6 = 0.008). CIDP, chronic inflammatory demyelinating polyradiculopathy; CMT, Charcot–Marie–Tooth disease; CSA, cross-sectional area; CMAP, compound muscle action potential; NCV, nerve conduction velocity.

## Data Availability

The data used to support the findings of this study are available from the corresponding author upon request.

## Supplementary Materials

The following supporting information can be downloaded at: https://www.mdpi.com/article/10.3390/medicina59071192/s1, Table S1: Correlation and comparison between ultrasound measurements in the entire cohort. Ultrasound measures did not differ between the left and right limb; Table S2: Correlation and comparison between nerve conduction study—tibial nerve. Electrophysiology measures did not differ between the left and right limb; Table S3: Subtype ALS comparison ultrasound data. ALS subtypes (bulbar vs. limbs; upper limb vs. lower limb) show no differences for ultrasound parameters; Table S4: Group comparison ultrasound data in CMT 1A. Comparable results with the total CMT cohort; Table S5: Correlation ultrasound data vs. clinical scores in CMT 1A. Comparable results with the total CMT cohort; Table S6: Correlation ultrasound data vs. nerve conduction study—tibial nerve in CMT 1A. Comparable results with the total CMT cohort; Figure S1: Box plots ALS—Blood–nerve barrier leakage. Shows the imaging parameters (CSA and microvascular blood flow) in ALS patients with blood–nerve barrier leakage against those without leakage; Table S7: Subgroup comparison clinical scores—ALS. Clinical ALS subgroups show no differences for imaging parameters; Table S8: Subgroup comparison clinical scores—CIDP. Clinical CIDP subgroups show no differences for imaging parameters; Table S9: Subgroup comparison clinical scores—CMT. Clinical CMT subgroups show no differences for imaging parameters.
