# Supplementary material for: Peripheral Nerve Ultrasound for the Differentiation between ALS, Inflammatory, and Hereditary Polyneuropathies
Source: Medicina (Kaunas). 2023 Jun 24;59(7):1192. doi: 10.3390/medicina59071192 (PMC10383275; doi:10.3390/medicina59071192)
Supplement: Supplementary file 1 [file medicina-59-01192-s001.zip › medicina-2418617-supplementary.pdf]

Table S1. Correlation and comparison between ultrasound measurements in the entire cohort.

**Total cohort (*n* = 36)**	**Correlation**	***p*-value**
CSA tibial nerve left –<br>CSA tibial nerve right	0.912<br>(< 0.001)**	Z = -1.440;<br>*p* = 0.150
Microvascular blood flow left –<br>Microvascular blood flow right	0.540<br>(< 0.001)**	Z = -1.645;<br>*p* = 0.100
CSA tibial nerves –<br>Microvascular blood flow	0.375<br>(0.024)**	---

Data presented as Spearman's rank correlation coefficient (rho) (*p*-value), ***p*-value considered significant after Bonferroni adjustment (ie, $p \leq 0.05 / 2 = 0.025$). CSA, Cross sectional area.

Table S2. Correlation and comparison between nerve conduction study – tibial nerve.

	**CIDP (*n* = 5)**		**CMT (*n* = 5)**	
	**Correlation**	***p*-value**	**Correlation**	***p*-value**
CMAP ankle left–<br>CMAP ankle right	1.000<br>(< 0.001)**	Z = -1.604;<br>*p* = 0.109	0.900<br>(0.037)	Z = -0.944;<br>*p* = 0.354
CMAP popliteal fossa left –<br>CMAP popliteal fossa right	1.000<br>(< 0.001)**	Z = -1.633;<br>*p* = 0.102	-0.700<br>(0.188)	Z = -0.135;<br>*p* = 0.893
NCV left –<br>NCV right	0.959*<br>(0.010)**	T(4) = 0.482;<br>*p* = 0.655	-0.981*<br>(0.003)**	T(4) = -0.110;<br>*p* = 0.917
CMAP ankle –<br>CMAP popliteal fossa	0.973<br>(0.005)**	Z = -1.604;<br>*p* = 0.109	1.000<br>(< 0.001)**	Z = -2.023;<br>*p* = 0.043

Data presented as Spearman's rank correlation coefficient (rho) (*p*-value), *Pearson correlation coefficient (r) (*p*-value), ***p*-value considered significant after Bonferroni adjustment (ie, $p \leq 0.05 / 4 = 0.0125$); CIDP, Chronic inflammatory demyelinating polyradiculopathy; CMT, Charcot-Marie-Tooth disease; CMAP, compound muscle action potential; NCV, nerve conduction velocity.

Table S3. Subtype ALS comparison ultrasound data.

**US data**<br>**ALS (*n* = 11)**	**ALS bulbar**<br>**(*n* = 3)**	**ALS limbs**<br>**(*n* = 8)**	***p*-value**
CSA<br>(mm^2)	14.06±0.82<br>(13.39-14.97)	10.94±3.07<br>(5.82-15.47)	T(9)=1.681;<br>p=0.127
Microvascular blood flow	0.0138±0.0131<br>(0.00-0.03)	0.0091±0.0092<br>(0.00-0.02)	T(9)=0.675;<br>p=0.516
US data<br>ALS limbs (*n* = 8)	ALS lower limb<br>(*n* = 2)	ALS upper limbs<br>(*n* = 6)	*p*-value
CSA<br>(mm^2)	12.32±4.46<br>(9.16-15.47)	10.49±2.67<br>(5.82-13.21)	T(6)=0.703;<br>p=0.508
Microvascular blood flow	0.0172±0.0042<br>(0.01-0.02)	0.064±0.0090<br>(0.00-0.02)	U=2.000;<br>Z= -1.341;

p=0.286

Data presented as mean ± standard deviation, (range); US, ultrasound; ALS, Amyotrophic lateral sclerosis; CSA, Cross-sectional area. T-test and Mann-Whitney-U-Test were used. Significance was set at $p \leq 0.05$.

Table S4. Group comparison ultrasound data in CMT 1A.

US data	ALS (n = 11)	CIDP (n = 5)	CMT 1A (n = 4)	CON (n = 15)
CSA (mm²)	11.79 ± 2.97 (5.82-15.47)	20.97 ± 8.31 (9.69-33.05)	25.00 ± 2.83 (20.99-27.52)	9.15 ± 2.15 (5.61-13.80)
Microvascular blood flow	0.0104 ± 0.0099 (0.00-0.03)	0.0318 ± 0.0246 (0.00-0.06)	0.0100 ± 0.0093 (0.00-0.02)	0.0044 ± 0.0037 (0.00-0.01)

US data	p-value (ANOVA)	ALS vs. CIDP	ALS vs. CMT 1A	ALS vs. CON	CIDP vs. CMT 1A	CIDP vs. CON	CMT 1A vs. CON
CSA (mm²)	F(3) = 25.59; p < 0.001	< 0.001	< 0.001	0.554	0.759	< 0.001	< 0.001
Microvascular blood flow	F(3) = 7.592; p < 0.001	0.007	1.000	1.000	0.034	< 0.001	1.000

Data presented as mean ± standard deviation, (range); US, ultrasound; ALS, Amyotrophic lateral sclerosis; CIDP, Chronic inflammatory demyelinating polyradiculopathy; CMT, Charcot-Marie-Tooth disease, CON, Controls; CSA, Cross-sectional area. One-way ANOVA and post-hoc Bonferroni were used. Significance was set at $p \leq 0.05$. Significant values are shown in bold.

Table S5. Correlation ultrasound data vs. clinical scores in CMT 1A.

Correlation	CMT 1A (n = 4)			
	CMTNS total	CMTNS leg score	MRC-sum-score	MRC-lower limbs
CSA	-0.395 (0.605)	-0.111 (0.889)	-0.135 (0.865)	-0.512 (0.488)
Microvascular blood flow	0.233 (0.767)	-0.219 (0.781)	-0.250 (0.750)	-0.216 (0.784)

Data presented as Pearson correlation coefficient (r) (p-value), **p-value considered significant after Bonferroni adjustment ($p \leq 0.05 / 8 = 0.00625$). CMT, Charcot-Marie-Tooth disease; CSA, Cross-sectional area; MRC-sum-score, Medical research council sum score; CMTNS, CMT-neuropathy score.

Table S6. Correlation ultrasound data vs. nerve conduction study – tibial nerve in CMT 1A.

Correlation	CMT 1A (n = 4)		
	**CMAP ankle**	**CMAP popliteal fossa**	**NCV**
CSA	0.204* (0.796)	0.400 (0.600)	0.693* (0.307)
Microvascular blood flow	-0.242* (0.758)	0.200 (0.800)	-0.669* (0.331)

Data presented as Spearman’s rank correlation coefficient (rho) (p-value), *Pearson correlation coefficient (r) (p-value), p-value considered significant after Bonferroni adjustment ($p \leq 0.05$ / 6 = 0.008). CMT, Charcot-Marie-Tooth disease; CSA, Cross-sectional area; CMAP, Compound muscle action potential; NCV, Nerve conduction velocity.

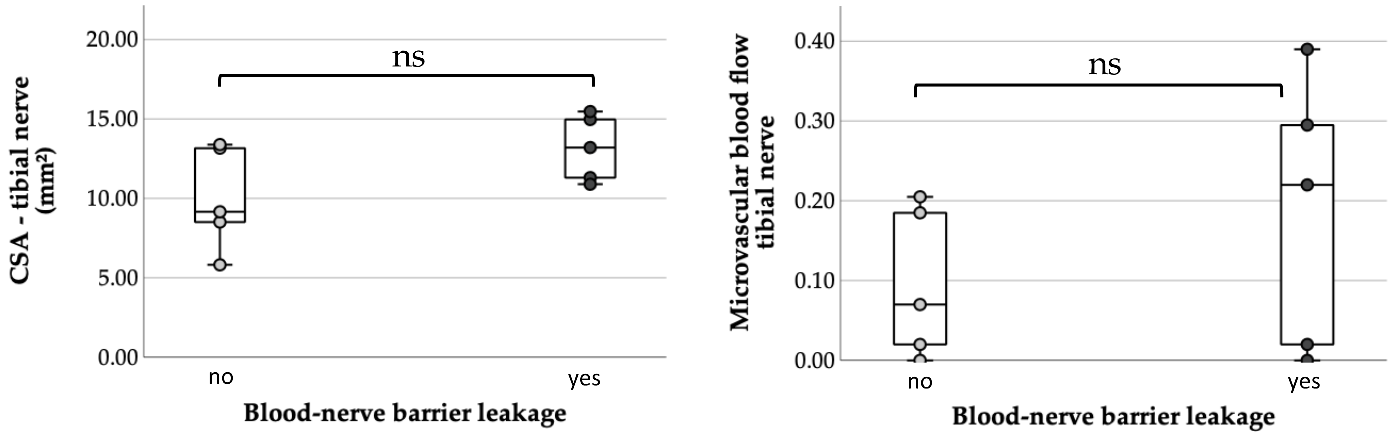

Figure S1. Box plots ALS – Blood-nerve barrier leakage.

A. Comparison of two ALS subgroups after division into present blood-nerve barrier leakage (yes) and intact blood-nerve barrier (no). The subgroup with blood-nerve barrier leakage shows not significant slightly larger CSA values. **B.** Illustration of microvascular blood flow without significant difference of ALS subgroups with blood-nerve barrier leakage (yes) and intact blood-nerve barrier (no). Median (horizontal bars), 25th to 75th quartile (box), range (whiskers) and single values (dots) are shown, CSA, Cross-sectional area; CSF, cerebral spinal fluid; ns, not significant.

Table S7. Subgroup comparison clinical scores – ALS.

**ALS (n = 11)**	**ALSFRS/R total**			**ALSFRS/R gross-motor**		
	**severe (n = 8)**	**mild (n = 3)**	**p-value**	**severe (n = 6)**	**mild (n = 5)**	**p-value**
CSA (mm²)	11.26 ± 2.82<br>12.24<br>(5.82-13.81)	13.20 ± 3.51<br>1.97<br>(9.16-15.47)	T(9) = -0.958; p = 0.363	11.11 ± 3.11<br>12.24<br>(5.82-13.81)	12.61 ± 2.91<br>13.21<br>(8.52-15.47)	T(9) = -0.821; p = 0.433
Microvascular blood flow	0.0067 ± 0.0087<br>0.0021<br>(0.00-0.02)	0.0202 ± 0.0059<br>0.0202<br>(0.01-0.03)	U = 3.000; Z = -1.854; p = 0.085	0.0082 ± 0.0090<br>0.069<br>(0.00-0.02)	0.0130 ± 0.0116<br>0.0142<br>(0.00-0.03)	T(9) = -0.777; p = 0.457

Data presented as mean ± standard deviation, median (range). p-value considered significant after Bonferroni adjustment (ie, $p \leq 0.05 / 4 = 0.0125$). ALS, Amyotrophic lateral sclerosis; CSA, Cross sectional area; ALSFRS/R, ALS-Functional Rating Scale/revised.

Table S8. Subgroup comparison clinical scores – CIDP.

**CIDP (*n* = 5)**	**ONLS total**			**ONLS leg grade scale**		
	**severe (*n* =2)**	**mild (*n* = 3)**	***p*-value**	**severe (*n* = 2)**	**mild (*n* = 3)**	***p*-value**
CSA (mm²)	27.50 ± 7.85<br>27.50<br>(21.95-33.05)	16.62 ± 6.01<br>19.67<br>(9.69-20.49)	T(3) =<br>-1.785;<br>p = 0.172	27.50 ± 7.85<br>27.50<br>(21.95-33.05)	16.62 ± 6.01<br>19.67<br>(9.69-20.49)	T(3) =<br>-1.785;<br>p = 0.172
Microvascular blood flow	0.0241 ± 0.0342<br>0.0241<br>(0.00-0.05)	0.0370 ± 0.0231<br>0.0257<br>(0.02-0.06)	T(3) =<br>0.515;<br>p = 0.642	0.0241 ± 0.0342<br>0.0241<br>(0.00-0.05)	0.0370 ± 0.0231<br>0.0257<br>(0.02-0.06)	T(3) =<br>0.515;<br>p = 0.642

**CIDP (*n* = 5)**	**MRC-sum-score**			**MRC leg**		
	**severe (*n* = 2)**	**mild (*n* = 3)**	***p*-value**	**severe (*n* = 2)**	**mild (*n* = 3)**	***p*-value**
CSA (mm²)	27.50 ± 7.85<br>27.50<br>(21.95-33.05)	16.62 ± 6.01<br>19.67<br>(9.69-20.49)	T(3) =<br>1.785;<br>p = 0.172	27.50 ± 7.85<br>27.50<br>(21.95-33.05)	16.62 ± 6.01<br>19.67<br>(9.69-20.49)	T(3) =<br>1.785;<br>p = 0.172
Microvascular blood flow	0.0241 ± 0.0342<br>0.0241<br>(0.00-0.05)	0.0370 ± 0.0231<br>0.0257<br>(0.02-0.06)	T(3) =<br>-0.515;<br>p = 0.642	0.0241 ± 0.0342<br>0.0241<br>(0.00-0.05)	0.0370 ± 0.0231<br>0.0257<br>(0.02-0.06)	T(3) =<br>-0.515;<br>p = 0.642

Data presented as mean ± standard deviation, median (range). p-value considered significant after Bonferroni adjustment (ie, $p \leq 0.05 / 8 = 0.00625$). CIDP, Chronic inflammatory demyelinating polyradiculopathy; CSA, Cross sectional area; ONLS, Overall neuropathy limitation scale; MRC, Medical research council.

Table S9. Subgroup comparison clinical scores – CMT.

**CMT (*n* = 5)**	**CMTNS total**			**CMTNS leg**		
	**severe (*n* =2)**	**mild (*n* = 3)**	***p*-value**	**severe (*n* = 2)**	**mild (*n* = 3)**	***p*-value**
CSA (mm²)	24.26 ± 4.62<br>24.26<br>(20.99-27.52)	23.07 ± 4.65<br>25.23<br>(17.73-26.26)	T(3) =<br>-0.279;<br>p = 0.798	22.63 ± 6.92<br>22.63<br>(17.73-27.52)	24.16 ± 2.79<br>25.23<br>(20.99-26.26)	T(3) =<br>0.365;<br>p =<br>0.739
Microvascular blood flow	0.0081 ± 0.0037<br>0.0081<br>(0.00-0.01)	0.0083 ± 0.0119<br>0.0031<br>(0.00-0.02)	T(3) =<br>0.028;<br>p = 0.980	0.0043 ± 0.0017<br>0.0043<br>(0.00-0.01)	0.0190 ± 0.0110<br>0.0107<br>(0.00-0.02)	T(3) =<br>0.804;<br>p =<br>0.480

CMT (n = 5)	MRC-sum-score			MRC leg		
	**severe (n = 3)**	**mild (n = 2)**	**p-value**	**severe (n = 3)**	**mild (n = 2)**	**p-value**
CSA (mm^2)	22.08 ± 4.99<br>20.99<br>(17.73-27.52)	25.74 ± 0.72<br>25.74<br>(25.23-26.26)	T(3) = -0.981; p = 0.399	23.84 ± 5.32<br>26.26<br>(17.73-27.52)	23.11 ± 3.00<br>23.11<br>(20.99-25.23)	T(3) = 0.170; p = 0.876
Microvascular blood flow	0.0064 ± 0.0039<br>0.0055<br>(0.00-0.01)	0.0110 ± 0.0155<br>0.0110<br>(0.00-0.02)	T(3) = -0.522; p = 0.638	0.0102 ± 0.0102<br>0.0055<br>(0.00-0.02)	0.0054 ± 0.0076<br>0.0054<br>(0.00-0.01)	T(3) = 0.556; p = 0.617

Data presented as mean ± standard deviation, median (range). p-value considered significant after Bonferroni adjustment (ie, $p \leq 0.05 / 8 = 0.00625$). CMT, Charcot-Marie-Tooth disease; CSA, Cross sectional area; CMTNS, CMT-neuropathy score; MRC, Medical research council.
